# Recent insights into noncanonical 5′ capping and decapping of RNA

**DOI:** 10.1016/j.jbc.2022.102171

**Published:** 2022-06-21

**Authors:** Selom K. Doamekpor, Sunny Sharma, Megerditch Kiledjian, Liang Tong

**Affiliations:** 1 Department of Biological Sciences, Columbia University, New York, New York, USA; 2 Department of Cell Biology and Neuroscience, Rutgers University, Piscataway, New Jersey, USA

**Keywords:** RNA capping, RNA decapping, RppH, NudC, Nudt12, DXO, Rai1, ApaH, ADPRC, ADP-ribosyl cyclase, APB, acryloylaminophenyl boronic acid, dpCoA, dephospho-CoA, Dxo1, decapping exonuclease, m7G, N7-methylguanosine, NAD capSeq, NAD captureSeq, NAD-capQ, NAD-cap detection and quantitation, NAD-RNA, NAD-capped RNA, NCC, noncanonical cap, Nudix, nucleotide diphosphate linked to X, PPH, pyrophosphohydrolase, ppRNA, diphosphate RNA, pRNA, monophosphate RNA, snoRNA, small nucleolar RNA, SPAAC, strain-promoted azide–alkyne cycloaddition, UDP-Glc, UDP-glucose, UDP-GlcNAc, UDP-*N*-acetylglucosamine, XRN, exoribonuclease

## Abstract

The 5′ *N*^7^-methylguanosine cap is a critical modification for mRNAs and many other RNAs in eukaryotic cells. Recent studies have uncovered an RNA 5′ capping quality surveillance mechanism, with DXO/Rai1 decapping enzymes removing incomplete caps and enabling the degradation of the RNAs, in a process we also refer to as “no-cap decay.” It has also been discovered recently that RNAs in eukaryotes, bacteria, and archaea can have noncanonical caps (NCCs), which are mostly derived from metabolites and cofactors such as NAD, FAD, dephospho-CoA, UDP-glucose, UDP-*N*-acetylglucosamine, and dinucleotide polyphosphates. These NCCs can affect RNA stability, mitochondrial functions, and possibly mRNA translation. The DXO/Rai1 enzymes and selected Nudix (nucleotide diphosphate linked to X) hydrolases have been shown to remove NCCs from RNAs through their deNADding, deFADding, deCoAping, and related activities, permitting the degradation of the RNAs. In this review, we summarize the recent discoveries made in this exciting new area of RNA biology.

The majority of cellular RNAs, particularly mRNAs, undergo modifications at both their 5′ and 3′ ends. These terminal modifications of the RNAs are central to their biological function. Chemical modification or addition of nonencoded residues to the 5′ end of RNAs is referred to as “5′ capping.” In eukaryotes, a cotranscriptional and sequential enzymatic addition of a 5′–5′ linked *N*^7^-methylguanosine (m^7^G) to the 5′ end of a nascent RNA polymerase II transcript is an extensively characterized and prominent 5′ modification, known as the m^7^G cap (Fig. 1*A*) (1).

**Figure 1:**
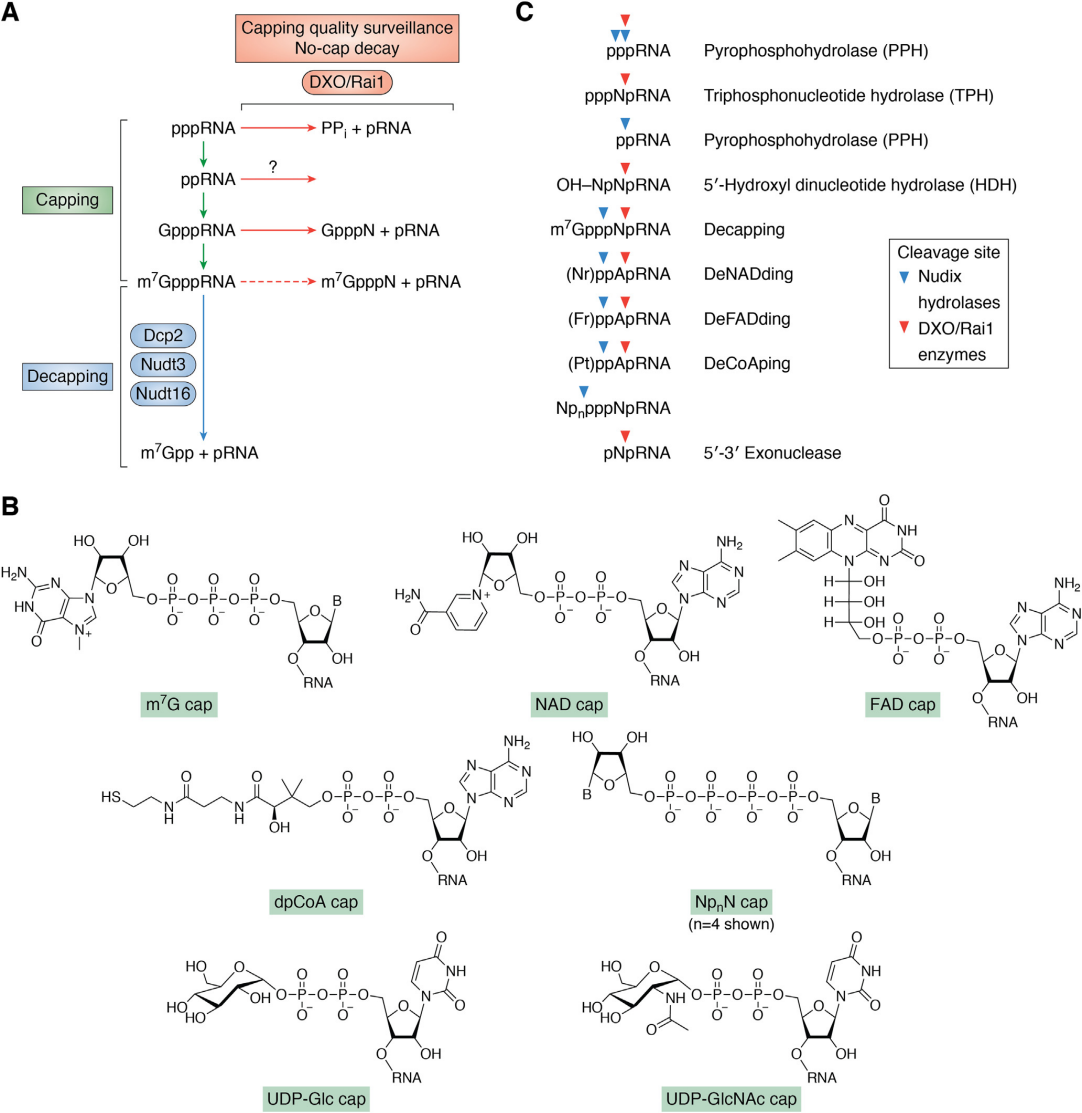
**The decapping activities of Nudix and DXO/Rai1 enzymes.***A*, schematic of the eukaryotic canonical, m^7^G capping (in *green*), and decapping (in *blue*) reactions. The enzymes that have canonical decapping activity are indicated. The 5′ capping quality surveillance reactions are also indicated (in *orange*). It is not known whether DXO/Rai1 enzymes also have activity toward ppRNAs. *B*, chemical structures of various caps (*B*, base). *C*, schematic of the various substrates of the decapping Nudix hydrolases and DXO/Rai1 enzymes. The cleavage sites of the Nudix hydrolases are indicated with the *blue arrowheads*, whereas those of the DXO/Rai1 enzymes are in *orange*. The cleavage site for the deNADding activity of XRNs is the same as that of DXO/Rai1 enzymes. The corresponding reactions are shown on the *right*. Fr, flavin ribitol of FAD; m^7^G, *N*^7^-methylguanosine; Nr, nicotinamide riboside of NAD; Nudix, nucleotide diphosphate linked to X; ppRNA, diphosphate RNA; Pt, pantetheine of CoA; XRN, exoribonuclease.

Historically, only eukaryotic transcripts were believed to contain 5′ modifications because analysis of bacterial RNA composition using one- or two-dimensional chromatography did not detect modification at the 5′ end (2, 3). Nonetheless, pioneering work in the late 1970s demonstrated that nucleotide metabolites (NAD and FAD), by virtue of their adenosine nucleotide moieties, could be utilized by *Escherichia coli* RNA polymerase as an initiating nucleotide for RNA synthesis *in vitro*, and the resemblance of 5′ NAD or FAD residue to the structure of mRNAs carrying the m^7^G cap was also noted (4). Approximately 2 decades later, nucleotide coenzymes, dephospho-CoA (dpCoA), NAD, and FAD, were shown to initiate transcription by the T7 class II promoters of T7 RNA polymerases *in vitro* (5), establishing the capacity of prokaryotic RNA polymerases to utilize adenosine-derived coenzymes as an initiating nucleotide *in vitro*. Moreover, bacterial RNA polymerases can also initiate transcription using highly abundant uridine-containing cell wall precursors, UDP-glucose (UDP-Glc) and UDP-*N*-acetylglucosamine (UDP-GlcNAc) (6), further establishing a possibility that nucleotide metabolites may decorate the 5′ end of bacterial RNA.

The first demonstration that noncanonical caps (NCCs) are indeed incorporated into RNA in cells was provided by a mass spectrometry (LC–MS)–based approach in 2009 (7, 8). NAD and dpCoA (and its acetyl, succinyl, or methylmalonyl derivative) were found linked to the 5′ end of RNAs in bacteria (*E. coli* and *Streptomyces venezuelae*). Collectively, these studies indicated that NCCs, in addition to the canonical m^7^G cap, may exist on mRNAs. Nevertheless, progress was hampered by the lack of methodologies that selectively identified specific RNAs harboring the nucleotide metabolite caps.

The first direct evidence demonstrating the existence of a noncanonical nucleotide metabolite cap at the 5′ end of RNA was provided in 2015 with the isolation and identification of NAD-capped RNAs (NAD-RNAs) in bacteria (9), followed by the demonstration that bacterial, mammalian, and mitochondrial polymerases can utilize nucleotide metabolites as noncanonical-initiating nucleotides *in vitro* and in cells (6, 10, 11). Reports of noncanonical metabolite caps in eukaryotes began in 2017 with their presence in budding yeast (12) and mammals (13), followed by plants (14). The field has since seen an explosion of progress in the identification of NCCs and the enzymes that can remove them (noncanonical decapping).

## Classification of noncanonical metabolite caps

Based on the caps identified to date in both prokaryotic and eukaryotic transcripts, the NCCs can be classified into two major classes—coenzyme caps and dinucleotide polyphosphate caps.

Coenzyme caps are derived from coenzymes that are complex organic molecules fundamental for many reactions in cells (15). Conspicuously, many coenzymes are derived from ribonucleotides, especially adenosine, such as NAD, FAD, coenzyme A, and S-adenosyl-methionine, and are viewed as the surviving vestiges of nucleic acid enzymes prior to the evolution of ribosomal protein synthesis (15). The known coenzyme caps are NAD, FAD, dpCoA, UDP-Glc, and UDP-GlcNAc (Fig. 1*B*). The best studied coenzyme caps are the NAD caps that will be described in more detail in this review.

The dinucleotide polyphosphate caps were identified in bacteria (16, 17, 18, 19). Dinucleotide polyphosphates (Np_n_Ns) were initially discovered as reaction intermediates of aminoacyl-tRNA synthetase–catalyzed reactions *in vitro* (20). Subsequent reports of the involvement of these molecules in stimulating DNA replication in quiescent human cells (21) and an approximately 100-fold increase in their concentration within 30 min following exposure to oxidative or heat shock stress emphasized their key physiological role as the signaling molecules that alert cells to the onset of specific metabolic stresses—hence named “alarmones” (22, 23). Although these molecules have been detected both in prokaryotes and eukaryotes, their precise function remained elusive. As exhibited for coenzymes caps, recent reports in bacteria from two independent groups (18, 24) demonstrated that RNA can be capped *in vivo* by several different Np_n_Ns and their methylated forms: Ap_3_A, m^6^Ap_3_A, Ap_3_G, m^7^Gp_4_Gm, Ap_5_A, m^6^Ap_5_G, m^6^Ap_4_G, m^6^Ap_5_A, and 2mAp_5_G (18). The type of capping is specific for the growth phase and can be induced by disulfide stress. Similarly, the recent demonstration that T7 RNA polymerase can incorporate thiamine adenosine triphosphate as a noncanonical-initiating nucleotide *in vitro* (25) and the elevation of this cofactor under metabolic stress in *E. coli* (26, 27) suggest additional classes of NCCs may yet be uncovered on cellular RNAs.

## Detection of noncanonical coenzyme caps

### NAD cap detection

#### NAD captureSeq

A major advance to the widespread study of NCCs came with the development of a chemoenzymatic-coupled next-generation sequencing–based method, NAD captureSeq (NAD capSeq) (9), for cataloging NAD-RNAs. NAD capSeq exploited a unique property of the *Aplysia californica* ADP-ribosyl cyclase (ADPRC) (28), which is capable of catalyzing transglycosylation reaction to replace nicotinamide with other *N*-heterocyclic nucleophiles including “clickable” alkynyl alcohols (9, 29). The alkynyl group replacing nicotinamide could then be conjugated to a biotin-azide in a copper-catalyzed cycloaddition reaction (30). The 5′-biotinylated transcripts are then enriched and subjected to next-generation sequencing. The NAD capSeq of *E. coli* transcripts further validated the existence of NAD-capped transcripts in bacterial cells and revealed that only a subset of small RNAs and mRNAs are NAD capped (9). Remarkably, subsequent independent NAD capSeq analyses of budding yeast (12), human (13), and plant (14) transcripts revealed that NAD capping is not only present in prokaryotes but also prevalent in eukaryotes.

Another version of NAD capSeq, named NAD tagSeq (31, 32), is based on a similar principle but instead of using biotin-azide for cycloaddition following the ADPRC reaction, the alkyne-functionalized NAD-RNA is ligated to a synthetic RNA (tagRNA) through an azide group at its 3′ end. Tagged RNAs are enriched using biotinylated DNA complementary to the tagRNA and sequenced directly using single-molecule RNA sequencing by Oxford nanopore methodology.

#### Strain-promoted azide–alkyne cycloaddition-NAD-Seq

A shortcoming of NAD capSeq is the use of copper ions as a catalyst for the cycloaddition reaction. Metal ions including copper can induce RNA fragmentation, which could impede the detection of low-abundance RNAs and result in bias toward shorter fragmented reads from the 5′ end of relatively abundant RNAs (33). This may account for the 5′ fragment bias of NAD-RNAs in mammals (13) and *Saccharomyces cerevisiae* (34). An alternative approach based on copper-free chemistry, strain-promoted azide–alkyne cycloaddition (SPAAC) reaction, was introduced to circumvent the use of copper ion catalysis (SPAAC-NAD-Seq) (33). The incorporated azide group is coupled to the highly reactive autocatalytic alkyne moiety of biotin-PEG4-dibenzylcyclooctyne, thus obviating the need for copper ions.

Another limitation of profiling NAD-RNAs by NAD capSeq in higher eukaryotes involves the residual activity of the ADPRC enzyme on m^7^G-capped RNAs, in particular with an adenosine as the first transcribed nucleotide (m^7^GpppA-RNAs) (33). Bis-adenine dinucleotide (Ap_2_A) and bis-hypoxanthine dinucleotide (Hp_2_H) also show affinity for the ADPRC active site, implying the possibility of additional purine substrates of this enzyme (35). Although the level of conjugation to non-NAD-capped RNA 5′ ends appears to be minute, the relative low abundance of NAD-RNAs presents a need for improved methodologies to ensure their exclusive detection. Depletion of m^7^G-capped RNAs by an anti-m^7^G cap antibody has been used as one of the tactics in SPAAC-NAD-Seq, although additional approaches to streamline NAD cap detection are needed.

### Non–sequence-based NAD cap detection

#### NAD-cap detection and quantitation

A streamlined and rapid method termed NAD-cap detection and quantitation (NAD-capQ) (36) to detect and quantitate levels of NAD caps in cells by replacing the mass spectrometry analysis with a versatile colorimetric assay can also be used to detect NAD caps. Similar to LC–MS–based approach to identify NAD caps (7), nuclease P1 activity is exploited to release the intact 5′ NAD from RNA. The released NAD is detected by a commercially available colorimetric assay. This assay is based on an enzymatic cycling reaction where NAD is reduced to NADH, which reacts with the probe to generate a product that absorbs light at a wavelength of 450 nm (36).

NAD-capQ was used to quantitate NAD-RNA levels in different organisms. Analysis of the levels in human cells demonstrated that NAD-RNAs respond to changes in cellular NAD concentrations, indicating that NAD capping provides a mechanism for human cells to directly sense and respond to alterations in NAD metabolism (36).

#### DNAzyme-coupled boronate affinity electrophoresis

Acryloylaminophenyl boronic acid (APB) PAGE is a modified Northern blot–based method to detect NAD-RNAs (11, 37). The method is based on the principle that the vicinal diol in 5′ NCCs (*e.g*., NAD, NADH, m^7^G) reduces mobility in PAGE supplemented with APB, which can be used to separate capped from uncapped RNAs (37). Because resolution at the nucleotide level is necessary to distinguish an RNA with or without an NAD cap, a short RNA molecule with a defined 3′ end is necessary. A DNAzyme-based cleavage, targeted by a complementary DNA, is used to generate 5′ fragments <150 nts that are resolved by APB PAGE and subsequently detected by Northern blot analysis (11). This approach was used to demonstrate the high prevalence of NAD-capped mitochondrial transcripts in *S. cerevisiae* (11). One limitation of this approach is that it is only suitable for abundant RNAs because its sensitivity is considerably lower than the PCR-based approaches.

### General NCC detection

#### Mass spectrometry–based methods

Mass spectrometry–based methods are the most versatile and sensitive methods for detecting NCCs in RNA. Initially used to demonstrate the presence of NAD and dpCoA caps on bacterial RNA (7, 8), it has since been used in multiple organisms (38). The basic principle is to isolate stringently purified total RNA, polyA + RNA, or size-fractionated RNA from noncovalently bound molecules, digest the RNA into nucleotides with nuclease P1, and analyze by mass spectrometry. Because the chemical bonding involved in 5′ capping (pyrophosphate or phosphate anhydride) is resistant to the phosphodiesterase activity of nuclease P1, 5′ NCC moieties remain intact. These are then analyzed both qualitatively and quantitatively by LC–MS/MS.

A systems-level mass spectrometry–based technique to detect and quantify NCCs including NAD caps, CapQuant (38), was recently introduced. After P1 nuclease digestion, an additional HPLC cap enrichment step allows greater sensitivity and specificity, up to attomole range per microgram of RNA analyzed. CapQuant analysis of RNAs from purified dengue virus, *E. coli*, *S. cerevisiae*, mouse tissues, and human cells validated the presence and provided relative amounts of new 5′ cap structures in addition to NAD caps, including FAD, UDP-Glc, and UDP-GlcNAc caps (38), unraveling a hitherto uncharted realm of cellular metabolites in RNA metabolism.

#### CapZyme-Seq

An enzymatic approach to detect and quantify RNAs with NCCs termed CapZyme-Seq (39) was introduced as a high-throughput sequencing method that uses nontraditional decapping enzymes like NudC (9) and Rai1 (13) to detect noncanonical capped-RNAs. In this method, decapping enzymes are first used to decap the noncanonical capped-RNAs to 5′-monophosphate RNAs (pRNAs), which are next ligated to a specific adaptor and subjected to next-generation sequencing. Based on the specificity of the decapping enzyme used, CapZyme-Seq could be utilized to detect NAD-, NADH-, dpCoA-, and FAD-capped RNAs. NudC was the first deNADding enzyme reported and can efficiently hydrolyze the NAD cap within its pyrophosphate to release nicotinamide ribose phosphate (Fig. 1*C*) (40). Rai1 and the DXO family of proteins remove the entire NAD moiety from the 5′ end of the RNA (13).

Despite the sensitivity of an RNA-Seq–based detection, diverse substrate selectivity of the decapping enzymes limits their versatility. Although NudC was initially reported to be a deNADding enzyme, it was subsequently shown to also function on canonical m^7^G-capped RNA, precluding its use for NAD caps (41). Rai1 lacks decapping activity on m^7^G-capped RNAs and possesses robust deNADding activity, making it a more suitable choice of enzyme (13, 42, 43). However, the data need to be interpreted cautiously because of the capacity of Rai1 to hydrolyze a battery of NCCs (NAD, FAD, dpCoA, and GlcNAc) (13, 39, 44). Perhaps a combinatorial approach with various NCC decapping enzymes in an additive and subtractive manor would best narrow down the precise cap on an RNA.

#### FAD-capQ

An approach analogous to NAD-capQ was developed to assess the presence of FAD-RNAs in cells, termed FAD-capQ (44, 45). FAD-capQ consists of a hydrolysis step to remove the intact FAD nucleotide metabolite from the 5′ end of FAD-RNAs combined with a fluorescent FAD detection system. Here, the NCC hydrolysis property of *Schizosaccharomyces pombe* Rai1 is leveraged to remove the FAD (as well as other NCCs) without subsequent degradation of the RNA body, which would otherwise generate a high background and preclude accurate interpretation of the fluorescence detection. *S. pombe* Rai1 releases intact 5′ FAD, which can be quantitated by a commercially available FAD fluorometric assay. The released FAD functions as an essential cofactor for an oxidase reaction and the corresponding fluorescence emission of the OxiRed probe (excitation/emission = 535/587 nm). The fluorometric assay parameters enabled detection of FAD in the femtomole range. Although a methodology has yet to be developed to precisely identify FAD-RNAs from cells, FAD-capQ was used to demonstrate a predominance of FAD-RNAs in RNA pools shorter than ∼200 nucleotides, indicating they may contribute to the function of regulatory RNAs (44).

## How are NCCs produced?

In contrast to canonical capping in eukaryotes (Fig. 1*A*) (46), various studies have demonstrated *ab initio* NAD capping mediated directly by RNA polymerases (5, 10, 15, 47), where instead of initiating with canonical ATP, RNA polymerases of both prokaryotes and eukaryotes, including mitochondrial RNA polymerase, initiate transcription with noncanonical metabolites. Although RNA polymerases usually favor ATP over NAD at A-initiating promoters, polymerases from mitochondria and some viruses like phage T7 have relatively higher incorporation rates of up to 40 to 60% for NAD or NADH relative to ATP (5, 10, 11). The enhanced utilization of NAD *versus* ATP by the mitochondrial polymerase may also explain the relatively higher NAD capping of mitochondrial transcripts (11). Moreover, there is a strong correlation between the promoter sequence and the efficiency of NAD incorporation, suggesting a contribution of sequence elements to NAD capping (39). A consensus promoter motif H_-3_R_-2_R_-1_A_+1_S_+2_W_+3_W_+3_ for *E. coli* RNA polymerase has been predicted for NAD capping, where H depicts higher propensity for A, T, and C; R having high rate of G and A; S having higher percentage of G and C; and W consisting predominantly of A and T (39). In addition to the promoter sequences, the Rif-pocket of RNA polymerase (6) is also vital for noncanonical capping in *E. coli*. Specific amino acid changes in the Rif-pocket of *E. coli* RNA polymerase, especially mutation of residue 516, exhibit a significant reduction in its ability to incorporate NAD (6).

Although *ab initio* NAD capping remains the only well-characterized mechanism for noncanonical capping, studies both in humans (13) and plants (48) suggest an alternative post-transcriptional mechanism as well. Support for this comes from the identification of NAD-capped intron-encoded small nucleolar RNAs (snoRNAs) (13). These RNAs are generated by exonucleolytic processing of intronic sequences, and the presence of snoRNAs with a 5′ NAD cap precludes their production through transcriptional initiation. The mechanism of post-transcriptional addition of an NAD cap is still unknown although several potential pathways were recently reviewed (49), highlighting potential alternative pathways for noncanonical RNA capping with abundant adenosine analogs.

## Functions of NCCs

### Functional role of NAD caps in RNA turnover

The canonical 5′ m^7^G cap is indispensable for efficient gene expression in eukaryotic cells (1). It fulfils a fundamental role in mRNA metabolism ranging from stabilizing the 5′ end of mRNAs to splicing, polyadenylation, nuclear export, and translation. Therefore, following validation of the existence of noncanonical RNA caps in different organisms, an important question arises regarding the functional nature of NCCs.

Functional characterization of NCCs is primarily restricted to NAD caps because these are the only class of caps that can selectively be identified and analyzed, which has revealed a clear influence on RNA turnover (9, 10, 13, 48). The most compelling evidence for a role of NAD caps in stability comes from transfection studies of NAD-RNA in eukaryotic cells (13). Contrary to the m^7^G cap, which bestows stability onto an RNA, the NAD-RNA was less stable than an uncapped RNA when electroporated into cells. These RNAs were more stable in cells devoid of the enzyme DXO, indicating that this protein hydrolyzes NAD caps (deNADding) in cells (13). Moreover, NAD-RNAs were abundant in DXO knockout cells indicating that the NAD cap tags an RNA for rapid decay and DXO contributes to this decay (13). Analyses in human cells revealed that both environmental stress and nutrient deprivation alter the relative amount of NAD-RNAs, and the NAD cap arguably plays a vital role in post-transcriptional reprogramming (41). Interestingly, the altered NAD-RNA levels under these stress conditions were a result of different mechanisms. Increased NAD-RNA levels following environmental stress were a consequence of reduced levels of the DXO deNADding enzyme following heat shock, whereas compromised deNADding activity of another deNADding enzyme, Nudt12, may account for the elevation under nutrient deprivation (41). Collectively, these data suggested that alteration of NAD-capped mRNA levels may provide a physiological link between the metabolic state of a cell and its mRNA composition (41).

A similar role for NAD caps serving as a destabilizing mark has also been reported in plants (48, 50, 51). In the absence of DXO1, the decay of NAD-RNAs in plants is likely mediated by the RDR6-dependent post-transcriptional gene silencing pathway (52). The NAD-capped transcriptome is remodeled in response to abscisic acid, although DXO1 is not required for this (48).

In contrast to its role in RNA decay in eukaryotes, NAD caps have been considered to fulfill the opposite function in prokaryotes, promoting RNA stabilization. However, this hypothesis is primarily premised on the observation that NAD-RNAs are more stable in strains lacking the NudC bacterial deNADding enzyme (9). NAD-capped transcripts were also found to manifest greater half-life *in vitro* compared with the uncapped triphosphate version of the transcript when treated with endonuclease RNase E and pyrophosphohydrolase RppH (9). The increased stability of NAD-RNAs in the absence of NudC supports a role for NudC in deNADding RNA in cells and thus promoting their decay in wildtype cells (9, 10). Considering this situation is exactly analogous to observations with the eukaryotic deNADding enzyme DXO, an alternative interpretation is that NAD caps mark bacterial RNAs for decay as well. NAD-RNAs are more stable and abundant in eukaryotic cells devoid of DXO, which indicates that NAD-RNAs are targeted for decay by this family of proteins (13). Therefore, the accumulation of NAD-RNAs in a NudC-disrupted strain indicates that the NAD cap is detected and degraded by NudC and actually serves as a degradation mark, even though the NAD cap does protect the RNA against degradation by RNase E (9). Additional studies assessing the stability of NAD-RNAs in wildtype cells containing NudC will be necessary to more definitively delineate whether NAD caps promote RNA stability or decay in bacteria.

### Potential role of NAD caps in mRNA translation

The significance of the m^7^G cap in mRNA translation raises the question whether NCCs also support translation. Initial studies using NAD-RNAs suggested they do not (13). Electroporation of an NAD-capped luciferase mRNA into human cells did not yield significantly different translation relative to a corresponding uncapped mRNA, whereas an m^7^G-capped mRNA was robustly translated as expected (13). Despite the limitation of this assay system that uses a transfected rather than endogenous mRNA to analyze translation efficiency, these data strongly suggest that NAD caps do not support translation. These findings were further corroborated in budding yeast cells, where NAD-RNAs were found not to support translation in cell-free extract prepared for *in vitro* translation and deNADding enzymes could be part of a surveillance mechanism for removing untranslatable NAD-RNAs (34).

A different scenario has been reported in *Arabidopsis thaliana* where NAD-capped mRNAs were isolated within the translating pools of ribosomes (14). Their presence in ribosomes suggests these transcripts may be translated. However, whether these mRNAs are serving a regulatory role or arose by ADPRC promiscuity remains to be determined (33). More detailed studies are needed to definitively address whether NAD caps can or cannot support mRNA translation.

### Contribution of NAD caps to mitochondrial function

NAD capSeq analysis of the budding yeast transcriptome demonstrated that a significant proportion of NAD-RNAs in this organism consisted of mitochondrially encoded transcripts (12). Direct quantitation of NAD- and NADH-RNAs by DNAzyme-coupled boronate affinity electrophoresis further demonstrated that remarkably up to 50% of two mitochondrially encoded transcripts, Cox2 and 21S, are NAD/NADH capped (11). Interestingly, the level of NAD/NADH capping by mitochondrial RNA polymerase was directly correlated with the metabolic state of the cells. Changing intracellular [NAD]/[NADH] ratios by growing the cells in a carbon source that supports fermentation or respiration resulted in changes in NAD and NADH capping of mitochondrial RNAs (11). The relatively high level of NAD capping in mitochondrial transcripts indicates they likely contribute to mitochondrial function.

To delineate the function of NAD-RNAs in *S. cerevisiae*, cellular proteins interacting with a 5′ NAD cap were identified using NAD cap RNA affinity purification (53). mRNA translation factors or proteins involved in cellular translation did not associate with the NAD cap in contrast to their efficient association with the m^7^G cap, supporting the lack of translation of NAD-RNAs in budding yeast. Remarkably, the prominent proteins associated with the NAD cap were RNA nucleases and not *bona fide* NAD cap-binding proteins, consistent with the NAD cap functioning as a tag to promote rapid RNA decay. In addition to Rai1, which is a well-characterized deNADding enzyme, the highly conserved 5′p-specific 5′-3′ exoribonucleases (XRNs), Xrn1 and Rat1, were found to associate with and hydrolyze NAD-RNA (53). Both Xrn1 and Rat1 are robust deNADding enzymes and, like DXO, remove the intact NAD from the RNA 5′ end and subsequently degrade the RNA (53).

Insights into the functional significance of Xrn1 and NAD caps were provided by the observation that in addition to its well-established cytoplasmic localization, Xrn1 also localizes within mitochondria (53). Moreover, a single amino-acid substitution (H41A) of Xrn1 resulted in a protein that still retained pRNA hydrolysis activity but lost deNADding activity. This mutation enabled the uncoupling of the two activities, and thereby the assessment of NAD cap contribution to mitochondrial function (53). In particular, the *xrn1-H41A* strain revealed that the previously characterized slow growth phenotype of *xrn1Δ* on nonfermenting sugar is primarily because of the loss of mitochondrial deNADding activity and not its 5′p-directed RNA decay. A ∼35% reduction in intramitochondrial NAD levels was observed in *xrn1-H41A* strain grown in yeast extract–peptone–glycerol, with glycerol as the nonfermentable carbon source). Because mitochondria are indispensable for respiration, a decrease in NAD levels in mitochondria led to a significant slow growth phenotype in yeast extract–peptone–glycerol media (53). Notably, mitochondria of budding yeast lack enzymes necessary for NAD biosynthesis and primarily rely on NAD imported from the cytoplasm (54). In addition, the availability of free NAD dictates levels of mitochondrial NAD-RNA synthesis (11). However, the full effect of Xrn1-H41A on mitochondrial NAD levels remains unclear because NAD-RNAs likely constitute <1% of total mitochondrial NAD levels (53). Whether this indicates cytoplasmic H41A deNADding may also contribute to mitochondrial NAD levels, or the pools of “free” NAD are a smaller fraction of the total NAD levels remains to be determined.

Collectively, the data support a role of Xrn1 in the turnover of mitochondrial NAD-RNAs that may contribute to mitochondrial NAD levels and cellular growth during respiration in budding yeast. A recent report in *A. thaliana* (48) reinforces the equilibrium between NAD caps and free NAD where stabilization of NAD caps led to a decrease in free NAD and compensatory increase in NAD production following stress. Whether NAD molecules stored in the 5′ end of RNA are used in adverse scenarios like nutrient starvation or quiescence in mammalian cells and the contribution of Xrn1 to NAD levels will be an important area of future exploration.

## Enzymes that remove NCCs

Dcp2, Nudt3, and Nudt16 are well-characterized decapping enzymes that remove the canonical m^7^G cap on RNAs in cells (Fig. 1*A*). They belong to the Nudix (nucleotide diphosphate linked to X) hydrolase family. Recent studies have shown that many Nudix enzymes, as well as the DXO/Rai1 enzymes, can remove NCCs from RNAs, giving rise to deNADding (removing NAD caps) (13), deFADding (removing FAD caps) (44), deCoAping (removing dpCoA caps) (44), and other activities (Fig. 1*C*). DXO/Rai1 enzymes are structurally unique among decapping enzymes and have neither sequence nor structural homology to Nudix enzymes. They have weak homology to D-(D/E)-X-K nucleases, present in some viruses and phage (55, 56).

### Nudix hydrolases

Nudix hydrolases are a large family of enzymes that are prevalent among eukaryotes, bacteria, and archaea. They possess pyrophosphohydrolase (PPH) activity and are often involved in nucleic acid biology (57). Nudix enzymes share a conserved 23 amino-acid Nudix motif (Nudix box) GX_5_EX_7_REX_2_EEXGU, where U is an aliphatic residue, that binds one or more divalent metal ions, which are essential for catalysis. *E. coli* has 13 Nudix proteins, including RppH and NudC, whereas mammalian cells have 22.

#### RppH

RppH plays a critical role in the 5′-dependent turnover of RNAs in bacteria, where its PPH activity converts 5′-triphosphate RNAs (pppRNAs) to pRNAs for subsequent decay by RNaseE (17, 58). *E. coli* RppH removes a pyrophosphate from pppRNA to produce pRNA (Fig. 1*C*). It can also eliminate a phosphate from 5′-diphosphate RNA (ppRNA) to produce pRNA, an activity that is faster by an order of magnitude (59). The latter activity appears to be the main role of *E. coli* RppH *in vivo* because ppRNAs, but not pppRNAs, are stabilized in its absence. This establishes ppRNA as a *bona fide* 5′ modification that can be regulated to impact gene expression. In comparison, *Bacillus subtilis* RppH catalyzes the consecutive removal of each terminal phosphate of ppp/ppRNA to produce pRNA. Both RppH orthologs require at least two single-stranded nucleotides at the 5′ end with a purine being preferred at the second nucleotide position. RppH activity can be further regulated by the DapF metabolic protein, which forms a 2:2 heterotetrameric complex with RppH and stimulates its activity (60, 61).

Besides ppRNA, RppH can also remove Np_4_N caps (16), producing pppRNAs (62). Np_4_N-capped RNAs are stabilized in *E. coli* cells in the absence of RppH during stress. Np_4_A is a better RppH substrate than pppRNAs and, similar to ppp/ppRNA substrates, RppH prefers purines within the RNA body (18, 62). RppH can also hydrolyze Np_n_N caps with different number of bridging phosphates and displays increasing activity with an increase in the number of phosphates present (18). The Ap_4_A hydrolase ApaH also has decapping activity against these caps, releasing ppRNAs or pRNAs depending on the length of the polyphosphate linker (16, 18).

*E. coli* RppH was initially shown to also display modest activity toward NADH-RNAs but no activity toward NAD-RNAs *in vitro* (9). However, later experiments showed that the enzyme has robust deNADding activity using a different RNA sequence and under different conditions (41, 47). This was further corroborated by the revelation that *B. subtilis* RppH also has manganese-dependent deNADding activity *in vitro*, and the NAD cap protects the RNAs against decay by the 5′-3′ exonuclease RNase J1 (47). RppH cleaves the pyrophosphate bond within NAD, producing NMP and pRNA (Fig. 1*C*).

**Figure 2:**
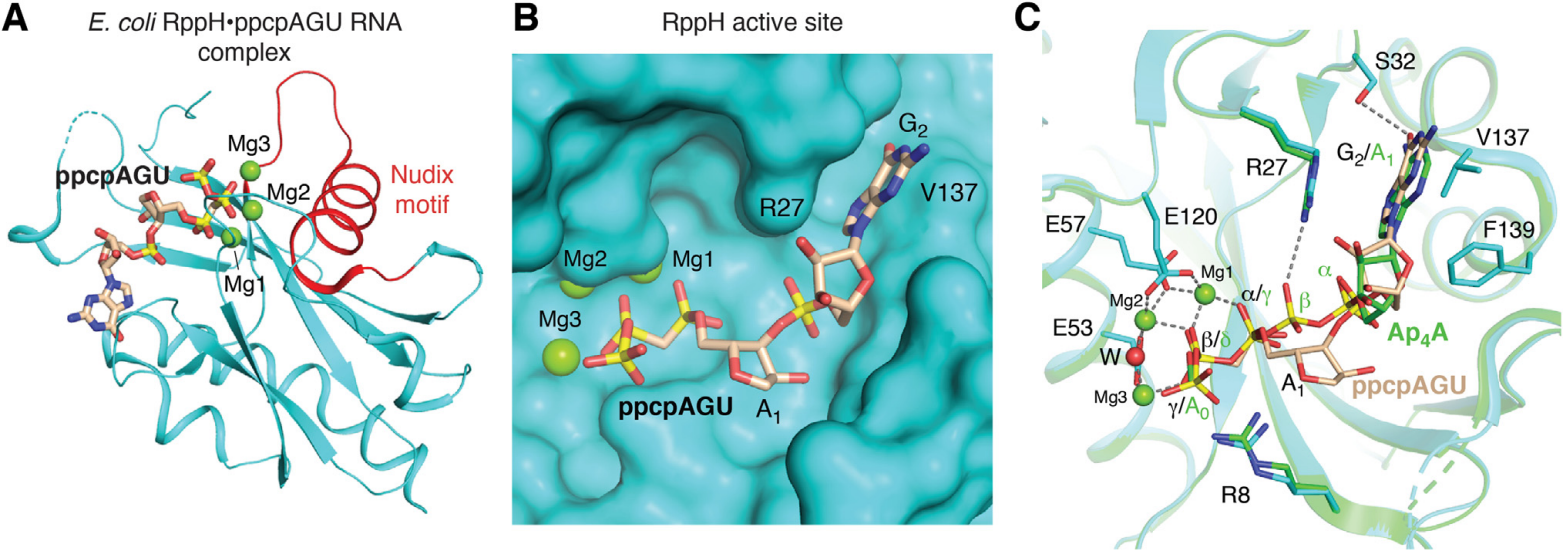
**Structures of RppH.***A*, structure of *Escherichia coli* RppH in complex with ppcpAGU RNA. Residues in the Nudix motif are shown in *red*. *B*, binding modes of the ppcpAGU RNA and Ap_4_A in the RppH active site. The adenine base of ppcpAGU was not observed in the crystal structure. *C*, molecular surface of RppH, showing the accommodation of the G_2_ base of ppcpAGU RNA in a surface cleft. Nudix, nucleotide diphosphate linked to X.

The catalytic mechanism of RppH was illuminated by the structure of *E. coli* RppH in complex with magnesium ions and a pppRNA (Fig. 2*A*) (63). RppH is a monomer and has the canonical Nudix fold, consisting of a seven-stranded mixed β sheet flanked by α helices. The active-site glutamates in the Nudix motif coordinate three magnesium ions, which bind the α and β phosphates and position the β phosphate for attack by a water molecule (Fig. 2*B*), generating pyrophosphate with pppRNA or phosphate with ppRNA as substrate. The second nucleotide of the substrate has cation–π interactions on one face of its base and hydrophobic contacts on the other, in a cleft that favors purines (Fig. 2*C*). The γ phosphate makes limited contacts with RppH, and it is unclear why ppRNA is favored over pppRNA for activity.

Ap_4_A binds the same site in RppH but in a distinct manner (62). The phosphate groups are shifted by two positions where the Ap4A δ and γ phosphates occupy the position of the 5′ppp β and α phosphates (Fig. 2*B*). Therefore, the δ phosphate undergoes nucleophilic attack, releasing pppRNA (Fig. 1*C*). The Ap4A α phosphate and adenine base are in the same position as the second nucleotide of pppRNA and can make similar contacts, with the adenine base also making stacking interactions.

Although the structure of RppH in complex with NAD-RNA is not known, it is likely that RppH uses a similar mechanism for its deNADding and PPH activities. The NAD pyrophosphate could bind the metal ions similar to the 5′ppp/pp α and β phosphates, consistent with the release of NMP as a product.

#### NudC

NudC was initially identified as a factor involved in recycling NADH to NMN and AMP (64). *E. coli* NudC lacks activity toward ppp/ppRNAs but was the first enzyme demonstrated to have activity toward NCCs (9, 10). NudC cleaves the pyrophosphate bond within NAD (Fig. 1*C*), and the pRNA product can be subsequently degraded by RNase E *in vivo* (9). NudC hydrolyzes NAD-RNA at a significantly higher rate than NADH/NAD alone (10) and also prefers NAD-RNA over NADH-RNA (9). NudC also has deCoAping activity, cleaving the pyrophosphate bond within dpCoA (10, 65) and is more active against this dpCoA-RNA than dpCoA alone (65).

**Figure 3:**
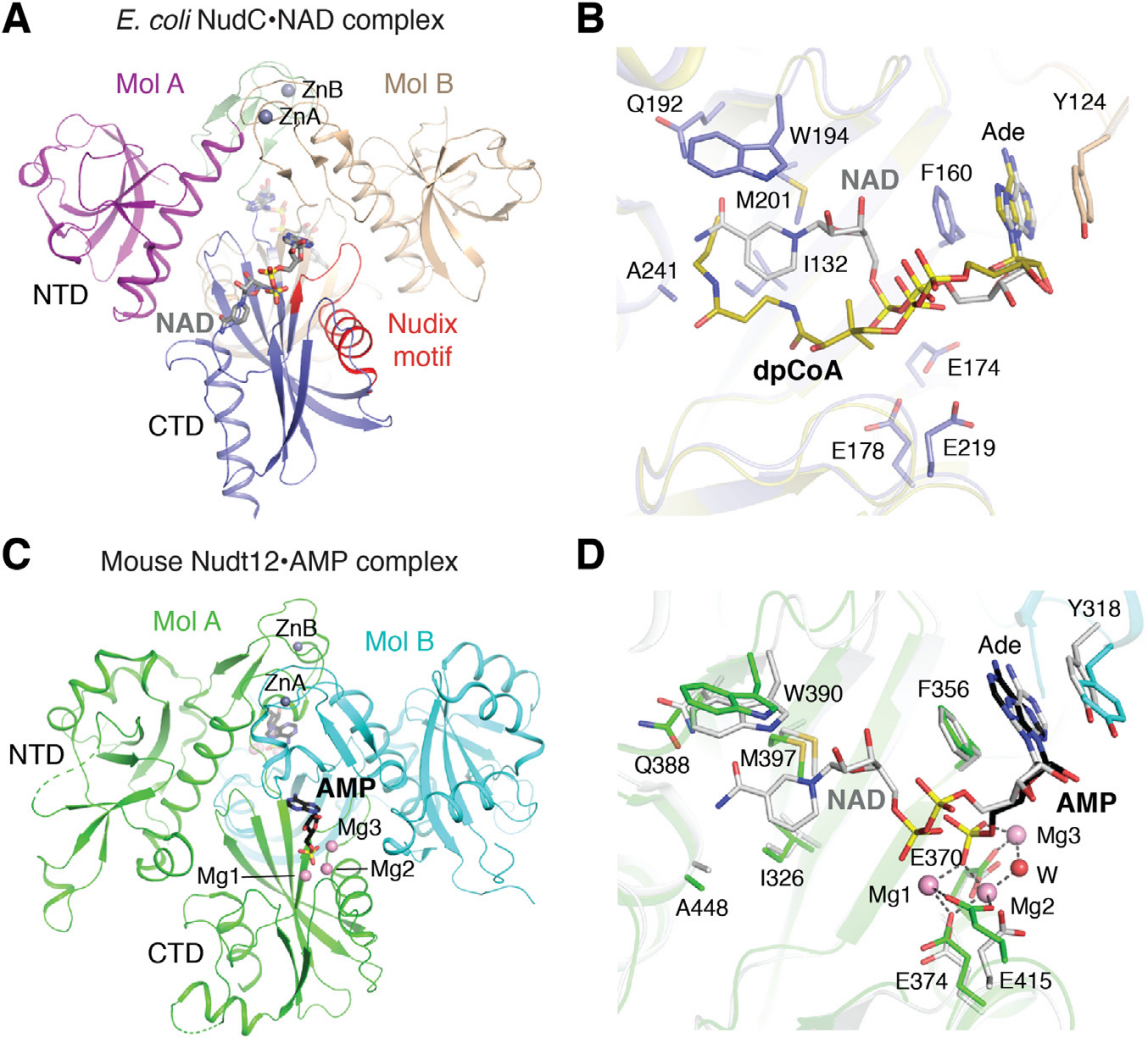
**Structures of NudC and Nudt12.***A*, structure of *Escherichia coli* NudC in complex with NAD. The domains of one monomer are given different colors. *B*, overlay of the binding modes of NAD and dpCoA in the active site of NudC. *C*, structure of mouse Nudt12 in complex with AMP and three magnesium ions. *D*, overlay of the binding modes of NAD in NudC and AMP in Nudt12.

NudC consists of two Nudix fold domains (N- and C-terminal domains) linked by a zinc-binding motif, although only the C-terminal domain contains the Nudix motif and is catalytically active. NudC forms a dimer, and the active site is located near the dimer interface (Fig. 3*A*) (40, 65, 66). NAD is bound in an extended conformation, with the adenine base recognized by π stacking with a Phe residue from one monomer and a Tyr from the other and the nicotinamide moiety buried in a hydrophobic pocket (Fig. 3*B*). The pyrophosphate group of NAD does not interact with the catalytic glutamate residues because of the absence of coordinating magnesium ions. However, comparisons to metal-bound structures of other Nudix enzymes suggest that NudC uses the same catalytic mechanism to hydrolyze NAD (also see Nudt12 later).

The structure of NudC in complex with dpCoA has also been determined (65). NudC employs a similar mechanism to recognize the AMP moiety of dpCoA, and the pantetheine group of dpCoA is accommodated in the same hydrophobic pocket that binds the NAD nicotinamide moiety (Fig. 3*B*). dpCoA also makes unique contacts to NudC, which is highlighted by the fact that mutation of certain residues within the hydrophobic pocket had different effects on the deNADding and deCoAping activities of NudC (65).

#### Nudt12

Nudt12 was the first eukaryotic Nudix protein shown to have deNADding activity, hydrolyzing the bond in the pyrophosphate (Fig. 1*C*) (41). It also has GpppN-RNA decapping and deCoAping activities *in vitro* but no deFADding activity. Nudt12 is a close homolog of bacterial NudC and was identified as a peroxisomal NAD/H diphosphatase (67, 68). Despite working in concert with DXO to deNAD RNAs in cells, Nudt12 preferentially targets a subset of RNAs that are distinct from DXO. In the absence of Nudt12, NAD-RNAs for histone and nuclear mRNAs coding for mitochondrial proteins were elevated, whereas NAD-capped sno/small Cajal body–associated RNAs are enriched in DXO knockout cells. The mechanism of this selection is not clear. Nudt12 interacts with bleomycin hydrolase, which does not alter its activity but localizes Nudt12 to cytoplasmic granules (69).

Nudt12 contains a unique N-terminal ankyrin repeat domain in addition to the two Nudix domains (41, 70). The ankyrin repeat domain is important for RNA deNADding activity but dispensable for hydrolysis of NAD itself. Insight into the deNADding mechanism of Nudt12 was revealed by the postcleavage structure of the Nudt12 catalytic domain in complex with AMP and three magnesium ions (Fig. 3*C*) (41). The AMP phosphate group contacts the magnesium ions and the conserved glutamate residues in the active site, with a water poised to attack the scissile phosphate (Fig. 3*D*). Comparison with NudC shows that NMN can be accommodated by Nudt12 in the analogous hydrophobic pocket. While the structure of Nudt12 in complex with dpCoA has not been determined, it is likely that dpCoA can also be accommodated in the active site.

#### Nudt16

Nudt16 was initially characterized as a U8 snoRNA decapping enzyme in *Xenopus laevis* and is better known for its involvement in canonical mRNA decapping in the cytoplasm (Fig. 1, A) (71, 72, 73), where it influences a subset of mRNAs compared with Dcp2 (71, 74). Recent studies have shown that Nudt16 can also remove NADH, NAD, dpCoA, and FAD caps *in vitro* (Fig. 1*C*), with NAD being preferred over NADH and dpCoA (45, 75).

**Figure 4:**
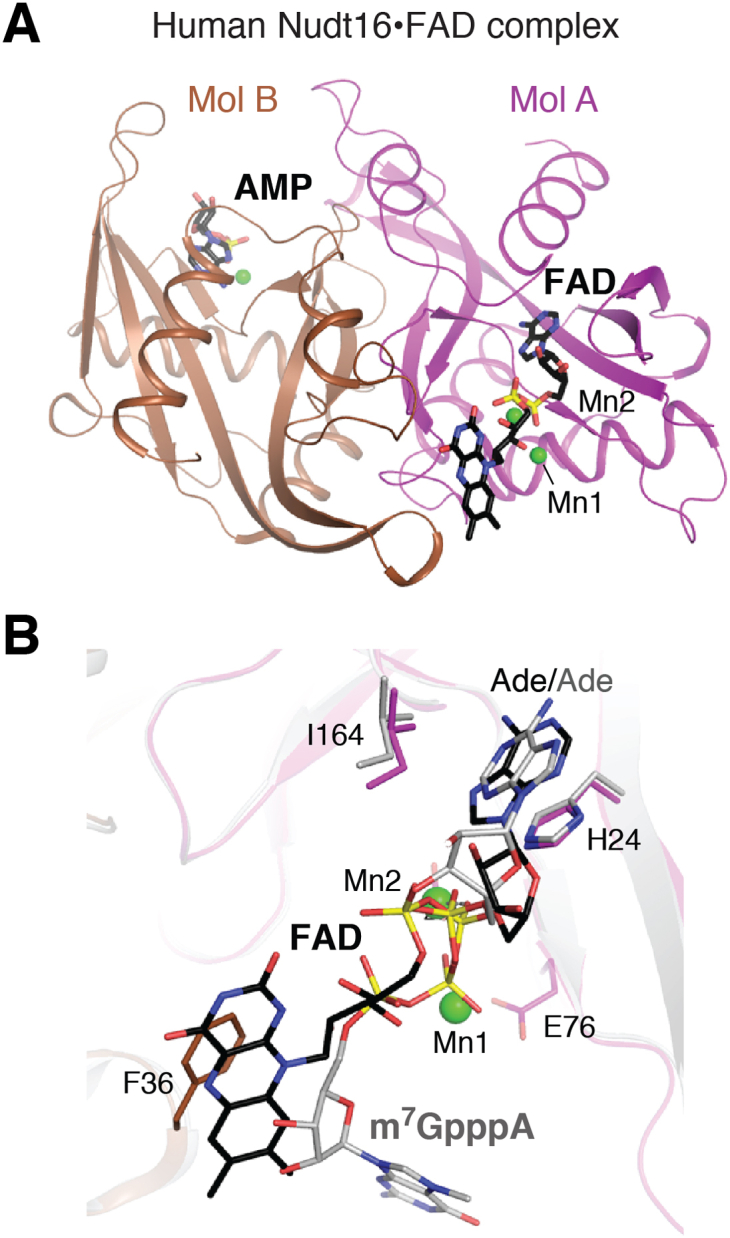
**Structures of Nudt16.***A*, structure of human Nudt16 in complex with FAD. *B*, binding modes of FAD in the active site of Nudt16 and m^7^GpppA in the active site of the *Xenopus laevis* homolog X29.

The structure of human Nudt16 in complex with FAD provides insight into how NCCs are recognized and hydrolyzed by this enzyme (45). Nudt16 has a single Nudix domain, and it also forms a dimer (Fig. 4*A*) but distinct from that of NudC and Nudt12. One monomer recognizes the adenine base, whereas the other monomer is involved in binding the flavin moiety of FAD through a π–π stacking interaction (Fig. 4*B*). A phosphate group contacts one of two manganese ions in the active site, whereas the ribitol group is mostly exposed to solvent. While the AMP segment of FAD is in a similar position compared with the equivalent group of other Nudt16 substrates, including m^7^GpppA, IMP, and ADPR, the flavin moiety is in a different position compared with the m^7^GpppA guanosine (Fig. 4*B*) (76, 77, 78). The large exposed surface of the active-site region likely explains the broad activities observed for Nudt12.

#### Nudt2

Nudt2 is also active on a broad range of substrates, being able to hydrolyze guanosine caps and pppRNAs (Fig. 1*A*), an activity that is important for initiating decay of viral RNAs with diverse sequences (79). This broad activity also extends to the NCCs, with Nudt2 showing deFADding and deCoAping activities *in vitro* (Fig. 1*C*). Interestingly, NAD-RNA is not a substrate for Nudt2. Despite its deFADding activity *in vitro*, Nudt2 knockout in cells does not increase FAD-RNA levels, as is the case with Nudt16 and DXO. This may be because FAD-RNA detection is limited to total short RNAs and Nudt2 acts on different FAD-capped RNAs. Alternatively, redundancies in deFADding activities could obscure the activity of Nudt2 in cells. Nudt2 also hydrolyzes Ap_4_A although it is not known if this also occurs in the context of Ap_4_A-capped RNA (80). Nudt2 is a monomer and likely has a similar catalytic mechanism as RppH.

#### Other Nudix decapping enzymes

The activities of all mammalian Nudix enzymes toward NCCs have been characterized through an extensive biochemical analysis (45). Only Nudt12 and Nudt16 have deNADding activity, whereas only Nudt2 and Nudt16 have deFADding activity. On the other hand, several Nudix enzymes, including Nudt2, Nudt7, Nudt8, Nudt15, Nudt16, and Nudt19, have deCoAping activity. This high degree of redundancy may explain the inability to detect dpCoA-RNA in eukaryotic cells. Nudt7, Nudt8, and Nud19 also hydrolyze dpCoA/CoA and their derivatives, and they have been confirmed or implicated as peroxisomal CoA PPHs. While Nudt19 can hydrolyze canonical caps *in vitro*, no such activity has been demonstrated for Nudt7 or Nudt8.

Nudix enzymes in other organisms have also been shown to hydrolyze NAD-RNA. Npy1 is the yeast homolog of NudC and was previously demonstrated to hydrolyze NAD (81) and FAD (82). Npy1 has deNADding activity and cooperates with Dxo1 and Rai1 to regulate the NAD-RNA levels (34), but it targets different NAD-RNAs, with mRNAs and small NAD-RNAs being enriched in its absence. In addition, Nudt19 from *Oryza sativa* has been shown to have deNADding activity *in vitro* (66).

## DXO/Rai1 enzymes—biochemistry

The structure of *S. pombe* Rai1, initially determined because it helped the crystallization of its binding partner Rat1, unexpectedly revealed a putative active site (42). A magnesium ion is coordinated by several conserved acidic side chains and waters in this site, suggesting a hydrolase activity. An RNA 5′ PPH activity was then discovered serendipitously for Rai1 (42). Further studies revealed its activity toward other intermediates of the capping process, with Rai1 being able to remove the entire unmethylated cap (GpppN) (43), distinct from the activity of the Nudix enzymes (Fig. 1*A*). On the other hand, Rai1 has no activity toward the mature m^7^G cap. This led to the identification of the first RNA 5′ capping quality surveillance mechanism (no-cap decay), with Rai1 being involved in the degradation of incompletely capped RNAs, which is confirmed by studies in budding yeast (43). These incompletely capped RNAs are protected against degradation by XRNs, and Rai1 removes the protecting group and produces pRNA for degradation.

The mammalian homolog of Rai1, DXO (then known as Dom3Z), also has PPH and decapping activities, although it is not sensitive toward the methylation status of the cap (Fig. 1*A*) (83). Interestingly, DXO also possesses distributive 5′-3′ exonuclease activity and thereby can contribute to the degradation of the RNA. This exonuclease activity was first discovered for the budding yeast Rai1 homolog, now named Dxo1 (decapping exonuclease) (55).

Following the discovery of NAD removal by *E. coli* NudC, DXO and Rai1 were the first eukaryotic enzymes shown to have deNADding activity (13). Similar to its decapping activity, the entire NAD cap is eliminated by DXO/Rai1, which is also distinct from NudC (Fig. 1*C*). Subsequently, DXO was also shown to have deFADding and deCoAping activities, with the entire FAD or dpCoA cap being removed (44).

Since the first report in 2009 (42), biochemical studies have revealed that the DXO/Rai1 enzymes possess a wide spectrum of catalytic activities, including PPH, decapping, deNADding, deFADding, deCoAping, 5′-3′ exonuclease, triphosphonucleotide hydrolase (releasing pppN from pppRNA) (84), 5′-hydroxyl dinucleotide hydrolase (releasing 5′OH-NpN from 5′OH-RNA) (85) activities, highlighting the remarkable versatility of these enzymes. At the same time, these enzymes have highly diverse activity profiles, with each enzyme having only a subset of these activities (84). For example, while *S. pombe* Rai1 has PPH activity but lacks 5′-3′ exonuclease activity, *Kluyveromyces lactis* Dxo1 lacks PPH activity but has robust 5′-3′ exonuclease activity (55). On the other hand, both *S. pombe* Rai1 and *K. lactis* Dxo1 have deNADding, deFADding, and deCoAping activities. With its 5′-3′ exonuclease activity, *K. lactis* Dxo1 can single-handedly decap and degrade RNAs with these NCCs.

## DXO/Rai1 enzymes–structure and mechanism

**Figure 5:**
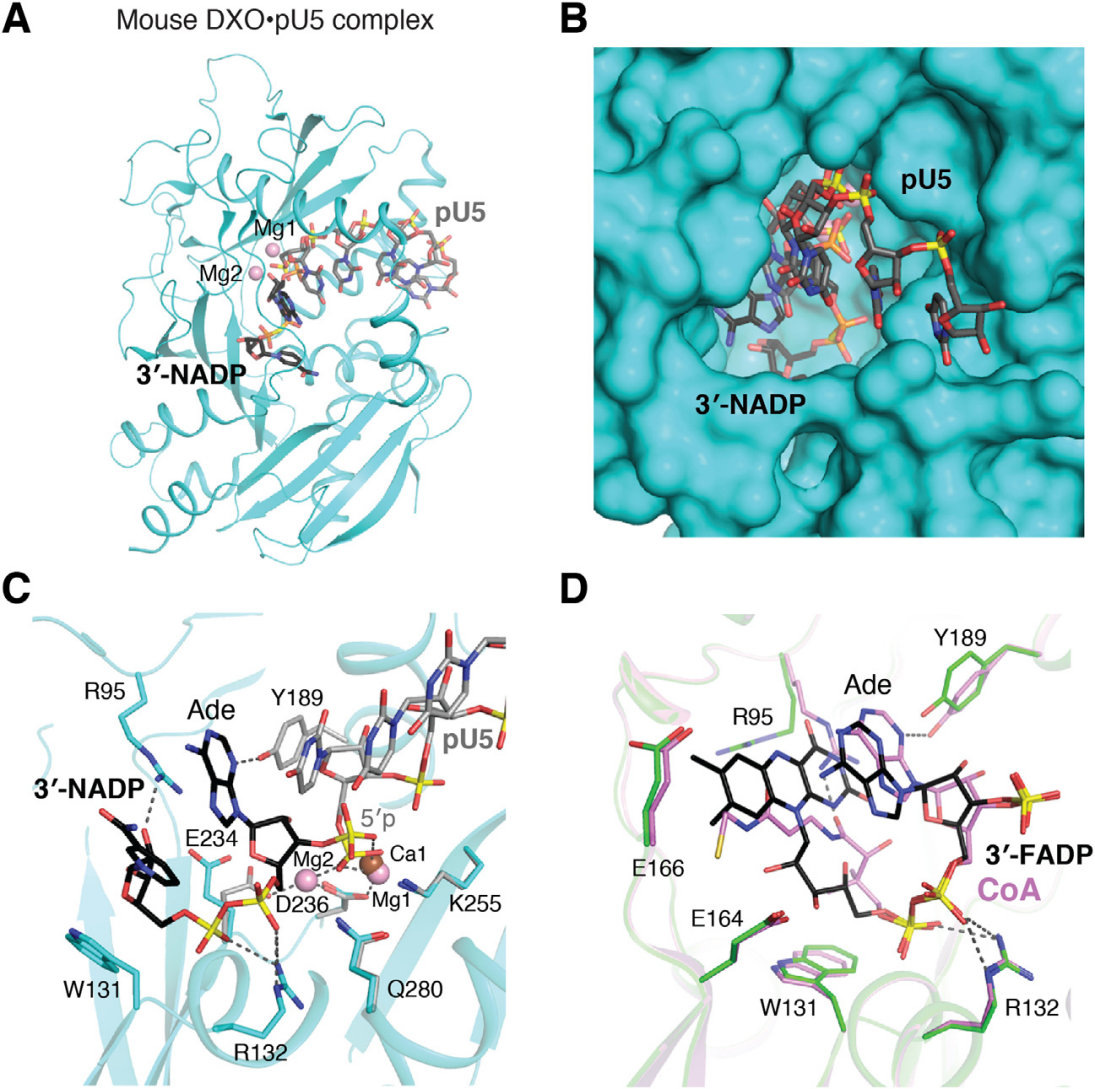
**Structures of DXO and Rai1.***A*, structure of mouse DXO in complex with pU5 oligo RNA product. The bound position of 3′ NADP is also shown. *B*, molecular surface of DXO showing the active-site pocket for RNA binding. *C*, binding modes of pU5 and 3′-NADP in the active site of DXO. *D*, binding modes of 3′-FADP and CoA in the active site of DXO.

DXO/Rai1 enzymes share six conserved sequence motifs (I–VI) that form the active site and are involved in metal ions and RNA binding (Fig. 5*A*) (84). Despite their diverse substrates and activities, the same active site is used for catalysis. This active site is located at the bottom of a deep pocket (Fig. 5*B*), consistent with the decapping and/or exonuclease activities of these enzymes. Two divalent ions (magnesium or manganese) participate in the catalysis (Fig. 5*C*), coordinated by negatively charged residues from motifs II, III, and IV (83). The metal ions bind and position the scissile phosphate for nucleophilic attack, and conserved residues from the other motifs recognize other regions of the substrate. For the 5′-3′ exonuclease activity, the 5′ phosphate is recognized by a conserved Arg from motif I (Arg132) near the base of the pocket, and ∼5 nts of the pRNA product can be accommodated in this pocket.

The structure of DXO in complex with an NAD-RNA mimic 3′-NADP and calcium ion demonstrated how NAD is recognized (Fig. 5, *A* and *C*) (13). The 3′ phosphate of 3′-NADP contacts one calcium ion occupying the metal ion–binding site, confirming that DXO releases the entire NAD cap. The AMP portion of NAD occupies a similar position as the 5′ nucleotide of an exonuclease substrate, whereas the NMN portion binds deeper in the pocket where there is weak conservation among DXO/Rai1 enzymes. The diphosphate is recognized by the same conserved Arg from motif I, and the NMN ribose is positioned against an aromatic residue from motif I.

The structures of DXO in complex with 3′-FADP or CoA illuminated how these much bulkier caps can also be DXO substrates (Fig. 5*D*). They bind in a folded conformation in a similar position as NAD, with their common pyrophosphate and adenosine groups binding in the same manner. The bulky flavin group is accommodated at the bottom of the pocket and is also π-stacked with the adenine base of FAD. For CoA, there is close contacts between the adenine base and the pantetheine group. Conformational changes of Tyr189 and a nonconserved arginine residue (Arg95) is observed depending on the presence of NAD, FAD, or CoA (Fig. 5, *C* and *D*).

Structures of Rai1 homologs in complex with RNA, 3′-NADP, and 3′-FADP have revealed the same organization of the catalytic residues (13, 44, 84). The nonconserved region at the bottom of the active-site pocket also undergoes conformational changes, to accommodate the different caps. This conformational flexibility is likely an important component of the versatility of these enzymes.

## Classes of noncanonical decapping enzymes

Depending on the mode of hydrolysis and whether it functions only on the NCC RNA or also on the nucleotide metabolite, the NCC decapping enzymes can be divided into four classes (Table 1). Class 1 NCC decapping enzymes consist of proteins that remove the intact nucleotide metabolite and include the DXO/Rai1 enzymes and the 5′-3′ exoribonucleases Xrn1 and Rat1. Class 2 NCC decapping enzymes cleave within the pyrophosphate of a nucleotide metabolite of both the free coenzyme as well as when conjugated to RNA. Members of this class of enzymes are NudC, Nudt12, and Nudt16. The third class of NCC decapping enzymes is represented by RppH, which can only hydrolyze the pyrophosphate linkage of the NCC when conjugated to RNA but not the free nucleotide metabolite. ApaH is in the fourth class. It has decapping activity toward dinucleotide polyphosphate caps and can also hydrolyze the dinucleotide polyphosphate itself. The structure of ApaH shares no homology with Nudix hydrolases, DXO/Rai1 enzymes, or XRNs.

**Table 1:** Classes of noncanonical decapping enzymes

Class	Enzyme	Substrate	Product
1	DXO Dxo1 Rai1 Xrn1 Rat1	XppApRNA	XppA + pRNA
2	NudC Nudt2 Nudt7 Nudt8 Nudt12 Nudt15 Nudt16 Nudt19	XppA or XppApRNA	Xp + pA or Xp + pApRNA
3	RppH	XppApRNA	Xp + pApRNA
4	ApaH	NppppN or NppppNpRNA	Npp + ppN or Npp + ppNpRNA

Noncanonical capped RNA or the free nucleotide metabolite are shown. The “X” can denote nicotinamide riboside, flavin ribitol, or pantetheine of NAD, FAD, or dpCoA, respectively, depending on the endogenous substrate of each enzyme.

## Future perspectives

Recent studies have established that NCCs are widely distributed in living organisms. However, the cellular functions of RNAs carrying NCCs are still poorly understood. NAD-RNAs can be individually identified through NAD capSeq, NAD tagSeq, or related technologies. In contrast, the identities of RNAs carrying the other NCCs are still not known. It will be important to develop technologies that would allow the sequencing and identification of RNAs carrying these NCCs, such as FAD-RNAs and dpCoA-RNAs. Knowing the identities of the RNAs may also help to unveil the functions of the various NCCs.

The studies of NAD-RNAs in mitochondria suggest the tantalizing possibility that these RNAs are a storage form of NAD. *S. cerevisiae* cells disrupted for Xrn1 mitochondrial deNADding activity exhibit a slow growth phenotype that is partially reversed by overexpression of the mitochondrial NAD transporter Ndt1. Despite the levels of NAD-RNAs constituting <1% of total mitochondrial NAD levels, these observations indicate a finely tuned equilibrium between NAD-RNAs and free NAD levels in cells that are controlled by deNADding enzymes. It will be interesting to determine whether under different physiological stresses, such as nutrient starvation or in minimalist cellular states like cellular quiescence, the contribution of NAD-RNAs will be even more pronounced.

Interestingly, Nudt2 is also implicated in cognition where biallelic disruption of the *NUDT2* gene leads to intellectual disabilities in affected individuals (86, 87, 88). The mechanism by which Nudt2 contributes to cognitive function will be an exciting future area of inquiry. In addition, it has been reported that an miRNA can base pair with the 5′ end of hepatitis C virus RNA and protect it from degradation by DXO (89).

While most NCCs are incorporated by RNA polymerases during transcription initiation, there is also evidence of recapping by NCCs. The strongest evidence for *de novo* NAD capping comes from the observations that snoRNAs that are generated from intronic RNA processing contain an NAD cap in human cells (13). Similarly, plant snoRNAs have also been reported (48). Importantly, unlike the possibility that a subset of eukaryotic RNAs reported to be NAD capped could have arisen by the promiscuity of ADPRC as described previously, the snoRNAs are not capped and would not have been identified through ADPRC nonspecific transglycosylation. The enzymes and pathways involved in NCC recapping are still not known.

The Nudix hydrolases and DXO/Rai1 enzymes have diverse activity profiles toward the mature m^7^G cap, incomplete caps, and NCCs. The molecular basis for this diversity is currently still poorly understood. The homologs of these enzymes are generally poorly conserved at the sequence level outside the catalytic machinery, and this sequence variability as well as conformational variability in the cap-binding region of the active site are likely important factors for their diverse activity profiles. Additional structural information, especially in complex with substrate or substrate analogs, as well as biophysical studies, will be needed to elucidate how this selectivity is achieved.

XRNs are newly identified deNADding enzymes (53). They have processive 5′-3′ exonuclease activity, and the active site recognizes the 5′p on the substrate (42, 90, 91, 92). The deNADding activity of XRNs removes the entire NAD, and how the NMN moiety is accommodated in the active site for this activity is currently not known.

Four classes of NCC decapping enzymes have been characterized so far (Table 1). It is likely that additional classes of decapping enzymes, as well as additional members of the classes, will be discovered in the future. It will be interesting to understand how (or whether) these diverse enzymes function together to regulate the 5′ cap on RNAs.

RNA 5′ capping and decapping had long been thought to be well understood. However, recent discoveries of a capping quality surveillance mechanism (no-cap decay) and the large number of NCCs have stirred excitement in this “back water.” Rapid progress has been made in the past few years, but many fundamental discoveries remain to be made in this new and burgeoning field of RNA biology.

## Conflict of interest

The authors declare that they have no conflicts of interest with the contents of this article.
